# Mitigating Burnout in an Oncological Unit: A Scoping Review

**DOI:** 10.3389/fpubh.2021.677915

**Published:** 2021-10-01

**Authors:** Rasheed Omobolaji Alabi, Päivi Hietanen, Mohammed Elmusrati, Omar Youssef, Alhadi Almangush, Antti A. Mäkitie

**Affiliations:** ^1^Research Program in Systems Oncology, Faculty of Medicine, University of Helsinki, Helsinki, Finland; ^2^Department of Industrial Digitalization, School of Technology and Innovations, University of Vaasa, Vaasa, Finland; ^3^Docrates Cancer Center, Helsinki, Finland; ^4^Department of Pathology, University of Helsinki, Helsinki, Finland; ^5^University of Turku, Institute of Biomedicine, Pathology, Turku, Finland; ^6^Department of Otorhinolaryngology – Head and Neck Surgery, University of Helsinki and Helsinki University Hospital, Helsinki, Finland; ^7^Division of Ear, Nose and Throat Diseases, Department of Clinical Sciences, Intervention and Technology, Karolinska Institute and Karolinska University Hospital, Stockholm, Sweden

**Keywords:** burnout—professional, mitigate, compassion fatigue, address, job satisfaction, oncology, artificial intelligence, stress

## Abstract

**Objectives:** The purpose of this study was to provide a scoping review on how to address and mitigate burnout in the profession of clinical oncology. Also, it examines how artificial intelligence (AI) can mitigate burnout in oncology.

**Methods:** We searched Ovid Medline, PubMed, Scopus, and Web of Science, for articles that examine how to address burnout in oncology.

**Results:** A total of 17 studies were found to examine how burnout in oncology can be mitigated. These interventions were either targeted at individuals (oncologists) or organizations where the oncologists work. The organizational interventions include educational (psychosocial and mindfulness-based course), art therapies and entertainment, team-based training, group meetings, motivational package and reward, effective leadership and policy change, and staff support. The individual interventions include equipping the oncologists with adequate training that include—communication skills, well-being and stress management, burnout education, financial independence, relaxation, self-efficacy, resilience, hobby adoption, and work-life balance for the oncologists. Similarly, AI is thought to be poised to offer the potential to mitigate burnout in oncology by enhancing the productivity and performance of the oncologists, reduce the workload and provide job satisfaction, and foster teamwork between the caregivers of patients with cancer.

**Discussion:** Burnout is common among oncologists and can be elicited from different types of situations encountered in the process of caring for patients with cancer. Therefore, for these interventions to achieve the touted benefits, combinatorial strategies that combine other interventions may be viable for mitigating burnout in oncology. With the potential of AI to mitigate burnout, it is important for healthcare providers to facilitate its use in daily clinical practices.

**Conclusion:** These combinatorial interventions can ensure job satisfaction, a supportive working environment, job retention for oncologists, and improved patient care. These interventions could be integrated systematically into routine cancer care for a positive impact on quality care, patient satisfaction, the overall success of the oncological ward, and the health organizations at large.

## Introduction

The oncologists are confronted with important decisions daily due to their dealings with patients with cancer (1). This makes this specialty inherently challenging as cancer is capable of inflicting devastation on the life of the person diagnosed with it. The individuals diagnosed with cancer usually experience psychological trauma, overwhelming emotions, and social and economic burden due to the effects of this deadly disease (2). As a result, oncologists are often exposed to long hours of direct patient care (3), medical counseling of the families of the patients, cumbersome electronic documentation, ever-changing medical environments, feeling of loss of control over daily responsibilities, and dissatisfaction in the provided resources by the health facilities to deal with emotional reactions of patients and their families (4, 5).

Despite the significant advances in the field of oncology, many patients with cancer still face long-suffering and die of the disease. This constantly exposes oncologists to difficult feelings of grief and compassion fatigue. At the same time, they have to use their cognitive and intellectual capacities to administer complex treatments to patients who are seriously ill. These aforementioned factors make them vulnerable to burnout syndrome (5). Following the first time that the term was described by psychologist Herbert Freudenberger, several attempts have been made to properly put this phenomenon into perspective (6–9). For instance, the WHO considered burnout as a syndrome that includes feelings of energy depletion, job dissatisfaction, and reduced professional performance (10). Likewise, it was construed as a form of chronic job stress that is characterized by three principal constructs, which include emotional exhaustion, cynicism and depersonalization, and reduced personal and professional efficacy (7, 11). Although, later definitions have eschewed personal and professional accomplishment as it was found that it overlaps with individual traits such as self-efficacy (12). Other forms of burnout manifestations include physical exhaustion, frequent oncology-related ethical mistakes, ineffectiveness, decreasing professional competence, unexplainable mood swing and absenteeism, and a sense of detachment toward colleagues and patients (13–15).

The challenge of burnout in oncology has a mean prevalence of 70% in Europe (16) and varies between 20 and 70% around the world (17, 18). Additionally, it is a multifaceted phenomenon with negative impacts on oncologists, colleagues, patients, and healthcare institutions (19). For oncologists, it affects personal well-being and increases the possibility of medical errors (17, 18). Consequently, these have profound effects on the patients as it affects their adherence to treatment recommendations and reduces the overall satisfaction of the medical care provided (20–23). Furthermore, these negative consequences underscore the long-term success of healthcare establishments (5, 24, 25).

In the absence of adequate well-being of oncologists, there is a significant chance for the oncologists to leave in search of a more conducive working environment or decrease the working hours (26, 27). Similarly, an increasing number of chemical dependence [alcoholism, drug addiction, and cigarette smoking (15)], and frequent disagreement between colleagues have been reported among oncologists (28). Also, the recent increase in suicide rates among junior doctors suggests the need to properly examine the issue of burnout in healthcare facilities (29). These observations are pointers that a holistic approach is important to understand the well-being of oncologists. Of note, in the current economic situation due to the COVID-19 pandemic, many institutions will have to deal with a decrease in staff numbers and other resources. It is expected that work may become increasingly stressful which may lead to burnout.

Therefore, understanding how to address oncologist burnout may offer an insightful step toward improving the quality of care offered to patients with cancer. As a result of this, we aim to conduct a scoping review of the published articles to identify the evidence-based approaches that focused on how to address and mitigate burnout in the oncology medical profession. Furthermore, we identify and analyze how artificial intelligence (AI) can assist to mitigate burnout in this field. The aforementioned research questions are important to map the current state of research in this subject area (oncology) when planning future research. Burnout in oncology was specifically considered as oncologists have been touted to be frequently affected by burnout syndrome and thus becoming one of the most vulnerable professionals (4, 30).

## Materials and Methods

### Search Protocol

**Figure 1:**
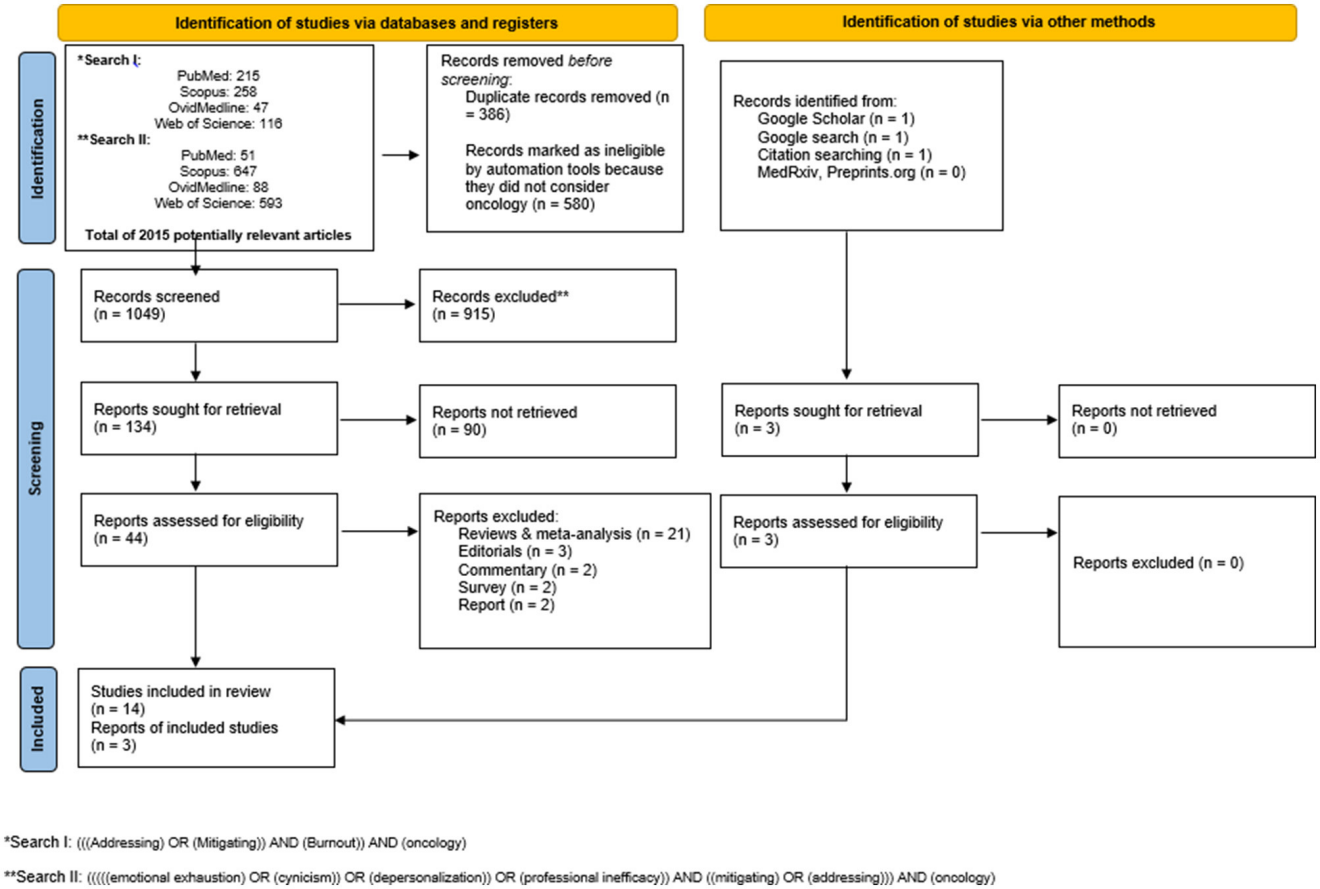
The Preferred Reporting Items for Systematic Review and Meta-Analysis (PRISMA) flow diagram highlighting the search strategy and the search results.

In this study, we systematically retrieved all studies that addressed burnout in oncology. The systematic search included databases of Ovid Medline, PubMed, Scopus, and Web of Science from inception until the end of July 2021. The framework that informed the search strategy was guided by the population (participants), concept, and context framework (PCC) (31). With the PCC paradigm, the questions of “who,” “what,” and “with what qualifiers” questions were answered. That is, the population (who: oncologists), concept (what: burnout), and context (what qualifiers: mitigate burnout) paradigm was used to formulate the research questions. Thus, the search approach was developed by combining search keywords: [(“addressing AND burnout AND oncology”) OR (“mitigating AND burnout AND oncology”)]. The search terms were extended to consider other related terms such as [(“emotional exhaustion” OR “cynicism” OR “depersonalization” OR “professional inefficacy” AND “mitigating” AND “oncology”)]. The search term extension was necessary to capture all possible studies. The retrieved hits were further analyzed for possible duplicates and irrelevant studies. Also, to further minimize the omission of any study, the reference lists of all the eligible articles were manually searched to ensure that all the relevant studies were duly included. In addition, experts were contacted and a Google search (Google Scholar) for relevant articles or PhD theses relating to this scoping review was done. To avoid selection bias due to selective publication, and most importantly, to enrich the scoping review process and reduce research waste, unpublished studies were also considered. The Preferred Reporting Items for Systematic Review and Meta-Analysis (PRISMA) was used in the searching and screening processes (Figure 1).

### Inclusion and Exclusion Criteria

All studies that had examined how to address or mitigate burnout in oncology were included in this study. However, studies that considered ways of mitigating or addressing burnout among radiation therapists, physician assistants, medical assistants, graduate medical trainees, radiologists, physicians, nurses, infusion center professionals, and nursing oncology were excluded. Additionally, studies that considered burnout interventions in other fields such as social sciences, psychology, environmental science, biochemistry, genetics, molecular biology, neuroscience, doctors, pharmacology, toxicology, and pharmaceutical; arts and humanities, business, management, and accounting were excluded. Similarly, to further enhance the quality of this study, comments, opinions, perspectives, editorials, reviews, abstracts only, and articles in languages other than English were excluded (Figure 1). As this study was aimed at a scoping review, a meta-analysis of the included studies was not performed.

### Screening

The screening was done in two distinct stages. In the first stage, two independent reviewers (RA and OY) used a data extraction sheet to examine the titles and abstracts of the retrieved and potentially relevant articles. The data extraction sheet was used to minimize the omission of possible eligible studies. The interobserver reliability was measured using Cohen's Kappa coefficient (κ = 0.83). In the second stage, possible discrepancies regarding the studies considered relevant were resolved by a consensus meeting and discussion between the two independent reviewers. The relevant information regarding the study characteristics of each of these potentially relevant articles was extracted (summarized in Table 1).

**Table 1:** The main findings from the included studies.

**References/type of study**	**Location**	**Title of the study**	**Size of participants (methodology)**	**Population**	**Main findings**	**Summary of mitigating burnout**
Le Blanc et al. (32)/original study	Netherlands	Take care! The evaluation of a team-based burnout intervention program for oncology care providers	• 664 participants (Questionnaire)	Oncologists	• Less depersonalization and emotional exhaustion were observed in wards where the burnout interventions were introduced • Burnout level is significantly related to job perception (meaningful job)	• Team focused participatory training intervention program (e.g., management support such as six sessions/month at 3 h for each session)
Italia et al. (15)/pilot study	Italy	Evaluation and art therapy treatment of the burnout syndrome in oncology units	• 65 participants (Maslach Burnout Inventory)	• Doctors and nurses (Group A) • Pediatric Oncology residents (Group B)	• The art intervention showed reduced level of burnout in Group B	• Recommendation of art treatment therapies
Bar-Sela et al. (3)/original study	Israel	“Balint group” meetings for oncology residents as a tool to improve therapeutic communication skills and reduce burnout level	• 17 participants (Prospective)	• Oncology residents	• Decrease in the incidence of burnout was observed	• Intervention method such as the Balint group meetings (for communication skills and strengthening doctor-patient relationships)
Moody et al. (33)/pilot study	United States and Israel	Helping the helpers: mindfulness for burnout in pediatric oncology- a pilot program	• 48 participants (Prospective)	• Pediatric Oncology	• The mindfulness-based course did not result in significant improvement in burnout, perceived stress, and depression	• Intervention program such as mindfulness-based course (MBC) could be useful for staff without burnout at baseline
Mukherjee et al. (34)/original study	United Kingdom	Staff burnout in pediatric oncology: new tools to facilitate the development and evaluation of effective interventions	• 32 participants (Interviews and surveys)	• Pediatric Oncology	• Two scales were created to facilitate the development and evaluation of effective intervention	• Interventions such as staff support, stress management and well-being interventions. • Evidence-based intervention such as Work Stressors Scale—Pediatric Oncology (WSS-PO) and Work Rewards Scale—Pediatric Oncology (WRS-PO)
Rasmussen et al. (35)/original study	Australia, United States, Netherlands, United Kingdom, Canada and Switzerland	Burnout among psychosocial oncologists: an application and extension of the effort-reward imbalance model	• 417 participants (Questionnaire)	• Psychosocial oncologists	• Higher effort and lower reward were significantly associated with greater emotional exhaustion, and not depersonalization • Overcommitment is strongly associated with both emotional exhaustion and depersonalization • Effort Reward Imbalance is partially supported for investigating burnout • Meaningful work is negatively related to emotional exhaustion and depersonalization	• Increasing self-efficacy • Positive changes to the oncological work environment • Increased rewards (money, praise, prize, better career opportunities) to the oncologists
He et al. (36)/original study	China	Dual role as a protective factor for burnout-related depersonalization in oncologists	• 131 (single role) and 168 (dual role) participants (Questionnaire)	• Oncologists (single role vs. dual role oncologists)	• Dual role oncologists (oncologists + psychosocial experience) showed less susceptibility to depersonalization, work-family conflict, and decision authority • Higher effort reward imbalance (ERI) predicted higher depersonalization in both dual role and single role oncologists • Overcommitment strongly associated with emotional exhaustion • Higher decision authority was associated with decreased emotional exhaustion • Work and Meaning inventory (meaningful work) is associated with decreased risk of depersonalization	• Psychosocial orientation to reduce depersonalization
Kavalieratos et al. (37)/original study	United States	“It is like heart failure It is chronic…and it will you”: A qualitative analysis of burnout among hospice and palliative care clinicians	• 20 participants (Interview)	• Palliative care clinicians	• Identified source of burnout include: • Increasing workload, tension between staff, regulatory issues • Variations between type of clinician and practice setting	• Individual (self-regulation, protective strategies, and protective strategies) solution • Interpersonal solution • Organizational change (change to working culture [frequent rotations on-and-off service], policy that encourages self-care of the staff, and regulation) solution.
Vetter et al. (38)/original study	United States	Resilience, hope and flourishing are inversely associated with burnout among members of the society for gynecologic oncology	• 374 gynecologic oncology	• Oncology	• Resilience • Hope	• Resilience, hope, flourishing and well-being metrics are inversely proportional to burnout • Male oncologists had higher level of hope, resilience, and well-being • Marital status is also an important factor
Richardson et al. (39)/original study	United States	Development of an “art of oncology” curriculum to mitigate burnout and foster solidarity among hematology/oncology fellows	• 16 participants (Prospective) • 26 fellows in total	• Oncologists • Fellows in Hematology-Oncology	• 93% respondents believed art of oncology (AOO) curriculum can address burnout • Work-life balance is associated with burnout • AOO can enhance solidarity among oncologists and fellow hematology-oncology	• Art of Oncology (AOO) curriculum
Kaimal et al. (40)/pilot study	United States	Outcomes of art therapy and coloring for professional and informal caregivers of patients in a radiation oncology unit: a mixed methods pilot study	• 34 participants (Prospective)	• Oncologists • Family caregivers	• Increase in Self-efficacy • Decrease in anxiety, perceived stress, and burnout	• Art therapy e.g., coloring or open studio art therapy (a 45-min session each) • Dedicated open studio space in oncological unit with art-making available
Weintraub et al. (41)/original study	United States	A cross-sectional analysis of compassion fatigue, burnout and compassion satisfaction in pediatric hematology-oncology physicians in the United States	• 363 participants (Survey)	• Hematology-Oncology	• Higher Burnout: • Compassion Fatigue • Administrative burden • Co-workers Lower Burnout: • Compassion satisfaction • Socializing Higher compassion satisfaction: • Exercise • Socializing • Talking with partners Lower compassion satisfaction: • Compassion Fatigue • Burnout • Emotional depletion • Administrative work • Inconvenient working environment	• Professional development in leadership • Communication • Conflict resolution • Team building and connectedness • Self-care
Royce et al. (42)/original study	United States	A burnout reduction and wellness strategy: personal financial health for the medical trainee and early career radiation oncologist	• NA	• Radiation oncologists	• Financial independent training	• The financial independent training can assist in improved quality of life for the oncologists through understanding student loans, debt management plan and independent of employment income
LeNoble et al. (43)/original study	United States	To address burnout in oncology, we must look to teams: reflections on an organizational science approach	• 409 participants (Prospective)	• Oncologists and Oncology provider	• Team-focused intervention led to higher level of team work and reduced levels of burnout. • It encouraged communication (interprofessional relationships), improved well-being and psychological safety • It positively affected the delivery of cancer care	• Team-focused burnout intervention approach
Turner et al. (44)/pilot	United States	The society of gynecologic oncology wellness curriculum pilot: A groundbreaking initiative for fellowship training	• 73 participants (Prospective)	• Oncology (Gynecologic)	• After the curriculum, the percentage of fellows that are comfortable to discuss wellness topic increased from 63 to 74%. Prior to the curriculum, 75% felt that they could identify symptoms of burnout or psychosocial distress.	• A structured curriculum aimed at promoting wellness amongst gynecology is imperative
Abusanad et al. (45)/original study	Saudi Arabia, Egypt, Sudan, Algeria, United Arab Emirates, Morocco, Yemen, Oman, Iraq, Syria, Lebanon, and Jordan	Burnout in oncology: Magnitude, risk factors and screening among professionals from Middle East and North Africa (BOMENA study)	• 1,054 participants (Prospective)	• Medical oncologists	• Hobby practicing • Oncology communication • Appreciate oncology work-life balance	• Having burnout skill, education, and support was found important to address burnout
Mascaro et al. (46)/original study	United States	Feasibility, Acceptability, and Preliminary Effectiveness of a Compassion-centered Team Intervention to Improve Clinical Research Coordinator Resilience and Well-being	• NA	• Oncologists (Clinical research coordinators)	• Compassion- centered, Team-based intervention Compassion-Centered Spiritual Health Team Intervention [CCSH-TI]	• The proposed team intervention may offer feasible, credible, and acceptable approach to providing resilience to oncology clinical research coordinators.

*Prospective, The study observes for outcomes of the effect of the introduction of certain interventions on some participants*.

*NA, Not available*.

### Quality Appraisal

The preliminary quality appraisal was done using the quality guideline for systematic review as recommended by the National Institute of Health Quality Assessment tools (47). The included studies in this review were subjected to four quality criteria informed by a similar study that used the same quality assessment tool (48). These criteria were modified to include design, methodology, interventions, and statistical analysis (Supplementary Table 1). The studies that showed reasonable quality (≥50%) were further subjected to the main quality assessment using the Agency for Health Research and Quality (AHRQ) tool. The AHRQ quality assessment tool was chosen as this scoping review aims at identifying means of burnout mitigation in oncology. The AHRQ has a total of 11 items for the methodological quality assessment (Supplementary Table 1) (49). The AHRQ has been scaled with score 0 for “NO” and “Unclear” and score 1 for “Yes.” An overall score of >8 indicated a “high” quality. Conversely, an AHRQ score between four and seven was defined as “medium” while a score of <4 was defined as “low” quality (49, 50). The score disagreements were resolved by consensus discussion between the two independent reviewers (RA and OY). The result of the quality assessment is presented in Table 2.

**Table 2:** Summary of quality assessment.

**References**	**AHRQ score**	**Quality interpretation**
Le Blanc et al. (32)	8	High
Italia et al. (15)	7	Medium
Bar-Sela et al. (3)	7	Medium
Moody et al. (33)	7	Medium
Mukherjee et al. (34)	8	High
Rasmussen et al. (35)	8	High
He et al. (36)	8	High
Kavalieratos et al. (37)	6	Medium
Vetter et al. (38)	8	High
Richardson et al. (39)	9	High
Kaimal et al. (40)	7	Medium
Weintraub et al. (41)	7	Medium
Royce et al. (42)	4	Medium
LeNoble et al. (43)	6	Medium
Turner et al. (44)	8	High
Abusanad et al. (45)	7	Medium
Mascaro et al. (46)	7	Medium

### Data Extraction

In each eligible study, the name of the first author, year of publication, country, the title of studies, results of the interventions, and summary of the strategies to address burnout in oncology were extracted (Table 1). A detailed explanation of the strategic interventions to mitigating burnout in oncology was discussed collectively in the discussion section.

## Results

### Results of the Database Search

The selection of eligible studies for this study is presented with the PRISMA flowchart (Figure 1). A total of 2015 hits were retrieved. After deleting duplicates (*N* = 386), irrelevant papers (*N* = 580), and exclusions (*N* = 915), we found 17 studies eligible to be included in this scoping review as shown in Figure 1 (3, 15, 32–46).

### Characteristics of Relevant Studies

All the articles included were published in the English language. Of the 17 included studies, 9 (52.9%) studies were conducted only in the United States (37–44, 46), 3 (17.6%) studies carried out in Europe (15, 32, 34), 2 (11.8%) studies each from Asia (3, 36), and 3 (17.6%) studies were from the international multicenter (33, 35, 45). From the included studies, 6 (35.3%) studies recommended individual interventions (how to address burnout) that include self-efficacy, self-regulation, and protective strategies for the oncologists (35–38, 41, 45). Furthermore, in addition to the individual intervention, 12 (70.5%) studies suggested organizational interventions that include organizing team-based participatory training such as group meetings, staff support, review policies, positive changes to the working environment, leadership and communication skills training for the staff, stress management, psychosocial education (mindfulness-based course, career development, resiliency training, and conflict resolution), motivational and encouragement (reward, prizes, and appreciation of efforts), and art treatment therapies (3, 15, 32–37, 39, 43, 44, 46) (summarized in Table 1). In terms of the quality assessment of the included studies, 7 (41.2%) of the included studies showed high-quality assessment scores. Conversely, 10 (58.8%) of the included studies were of medium-quality scores study (Table 2).

### Summary of the Findings From the Studies

**Figure 2:**
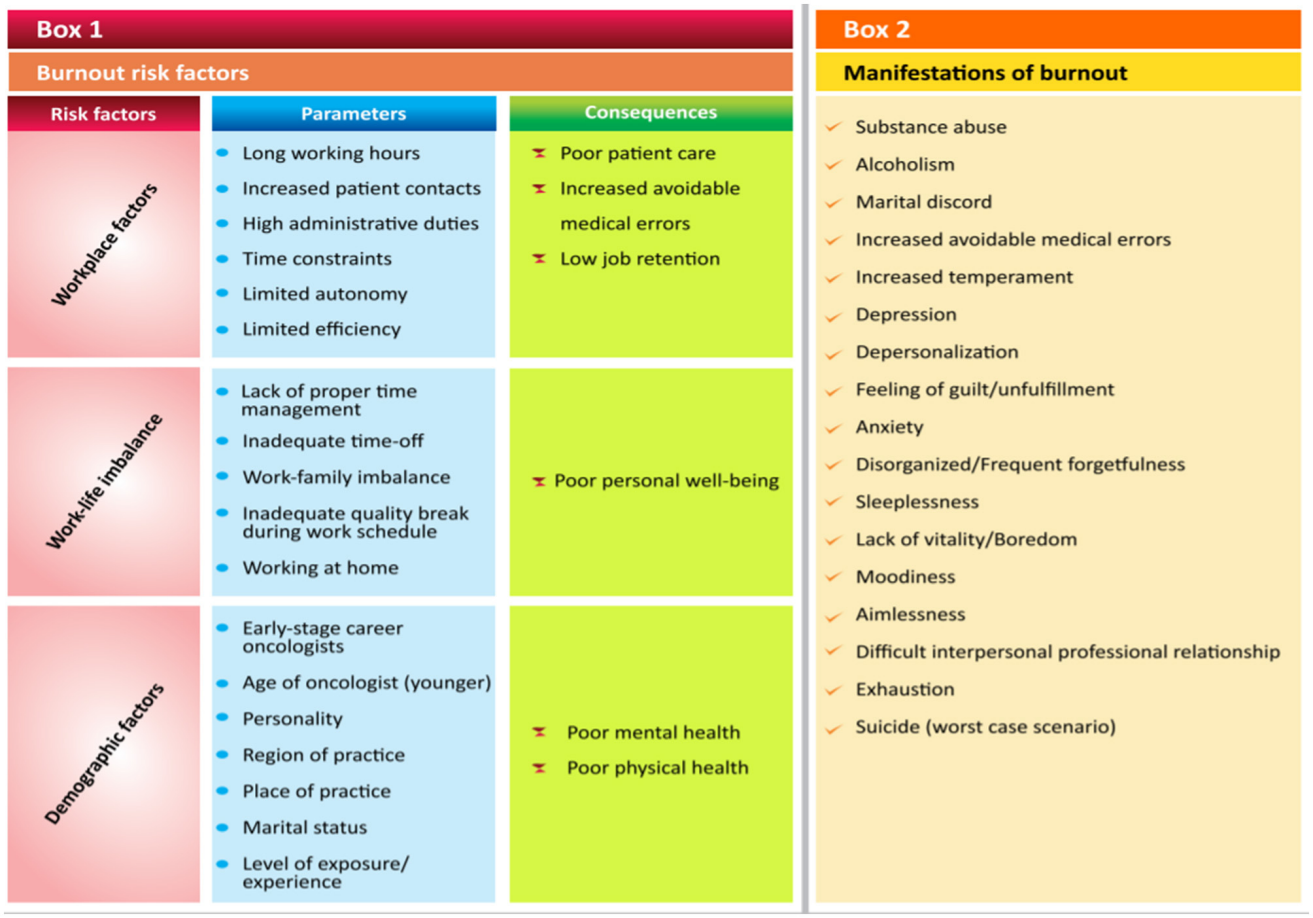
The prevalence, expression, and manifestations of burnout in the oncological unit.

The findings of these studies (summarized in Table 1) indicated that burnout is a substantial issue associated with oncology as a medical profession. The included articles contained several recommendations of interventions that could serve as the impetus for addressing and mitigating burnout in the oncological ward. These interventions were broadly categorized into seven divisions: individual, organizational, educational, art and entertainment, team-based, motivational, and disruptive technology. The risk factors found for the prevalence, expression, and manifestations of burnout in the oncological unit are presented in Figure 2 (Box 1).

The results also indicated that these interventions are poised to offer self-care, personal well-being (less depersonalization and emotional exhaustion), resilience and reduction to burnout, and job satisfaction and retention for the oncologists (3, 15, 32, 36, 39, 43). It was found that the burnout level is strongly associated with job satisfaction (32, 34). Considering the effort-reward imbalance model, higher efforts and a lower reward were reported to be associated with emotional exhaustion (35) and depersonalization (36). Meanwhile, overcommitment is strongly linked to both emotional exhaustion and depersonalization (35, 36). Having trained in psychosocial education was found to show a higher impact on work-family balance and reduce depersonalization (36). Similarly, creating a conducive working environment for meaningful work was associated with decreased risk of depersonalization (36).

## Discussion

In this scoping review, we examined the published studies on how to address or mitigate burnout in oncology as a medical profession. In terms of prevalence, specifically in the United States, it was reported that around 62% of the oncologists have reported experiencing specific symptoms of this burdensome phenomenon called burnout. In Europe and Australia, it varies significantly, ranging from 52 to 78% (5). Thus, without proper measures to address and mitigate burnout in oncology, the pool of available resources for proper cancer management may not achieve the desired objectives due to stress, depression, burnout, and tragically even suicide among the oncologists.

The stressor that causes burnout can be generic, work-related stressors such as monotonous, wearisome and unexciting tasks, significant workload, and poor interpersonal relationships with colleagues (34). In addition, excessive interruptions, meaningless documentation and regulatory specifications, cumbersome electronic health record (EHR) systems, and increasing pressure on the oncologists to attend to more patients without consideration on quality and oncologists-patients relationships are posited to putting the oncologists in morally compromising situations (51). Thus, increasing the possibility of stress and burnout (51). Also, there are job-specific stressors that are inherent to each job such as oncology (34, 52). An example of such stressors includes administering and managing complex treatment regimens for patients with cancer. Several work-related stressors in oncological settings have been published (27, 32, 53). Overcommitment has also posed as an individual stressor that is capable of causing emotional exhaustion and depersonalization which may eventually lead to burnout (35, 36).

Therefore, from the aforementioned, it becomes a shared responsibility between the oncologists and the organizations where they work to be determined in their approach toward reducing burnout and further cultivate resilience and career satisfaction (5, 54–56). This means that effective interventions that are poised to address and mitigate burnout may require that it is multifaceted, i.e., addressing organizational and personnel stressors that lead to burnout (33, 57). That is, individual oncologists are expected to identify burnout in themselves and their colleagues (5, 58). Similarly, organizations should be ready to offer systematic interventions (5, 58, 59).

Individual interventions for oncologists include self-efficacy, self-regulation, and protective strategies against stress and burnout (35–37, 41). However, the lack of confidentiality, stigmatization, and the trepidation of professional repercussions are some of the factors affecting medical staff such as the oncologists from help-seeking attempts on challenges such as behavioral health issues (burnout, depression, and suicide) (60). Therefore, the need for an inflection point to transparent and appropriate help-seeking behavior from the oncologists to address behavioral health issues such as burnout becomes imperative (60). To this end, the organization should provide the necessary help-seeking platforms and staff support (including psychological and bereavement support, working hours limitation, resiliency training, and team role clarifications) to avoid burnout (34).

Furthermore, it is important for organizations to carefully and critically develop an evidence-based and appropriate type of training for medical team leaders (24). It has been reported that good leadership skills and qualities are capable of reducing burnout, increases staff well-being, and position the organization in which they function for success (24). Also, the cordial relationship between the staff and their team leader or supervisor is a critical aspect of professional satisfaction (61). Therefore, the leadership types, skills, and qualities have a significant impact on well-being, degree of burnout, and professional satisfaction (24). The organizational factors that impact well-being include the nature of the practice environment (work-life balance), the level of autonomy offered to the staff, and the amount of workload assigned to the staff (54–56, 62). These interventions can replete oncologists and offset their emotional exhaustion (33).

Team-based interaction and group meetings improve communication skills, keep the team members informed, engage them through sharing ideas for improvements in their practice environments, discussions about career development, and provides constructive feedback to team members (24). Likewise, organizational interventions in the form of education, training, and short-term courses such as stress management, psychosocial education, mindfulness-based course, and conflict-resolution training. However, the level of stress and burnout within the oncological unit should be accessed before the introduction of any course or training for optimum results (33, 34). Similarly, the courses and training should be carefully structured to avoid adding another stress to the already busy professional schedule of the oncologists (33).

The use of context-specific measures to access work-related stressors and rewards as the reliance on generic measures may provide an incomplete picture of the level of stress among staff (34, 52). Thereby, this might lead to a recommendation of inappropriate burnout interventions. An example of the widely used measure is the effort-reward imbalance model. Of note, effort corresponds to the obligations and responsibilities that the employee is saddled with, while the rewards are not necessarily financial but may include esteem (respect), prizes and awards, job well-done appreciation, job security, and career opportunities and progression (34, 35). Thus, higher efforts and lower reward are strongly associated with emotional exhaustion (35) and both emotional exhaustion and depersonalization (36). Though, overcommitment predicts both emotional exhaustion and depersonalization. This is because, overcommitted individuals have a set of attitudes, behaviors, and emotions that make them strive to be approved (35, 63). To this end, they are usually prone to stress and burnout (35, 64).

It has been reported that the use of art treatment therapies in the form of coloring, dedicated open art studio with therapeutic support within the oncological unit, psychodrama, and relaxation increase the oncologist in self-care and decreases anxiety, perceived stress, and burnout in both oncologist and family caregivers (15, 40). Interestingly, participants with interest (or experience) in art-making were found to benefit more from this intervention (40). The motive behind the art-making intervention is to shift the attention of the stressed personnel away from their worries (40). Therefore, this brings to mind that various interventions should be offered on a personal level. This is because people benefit from different types of these interventions.

Although burnout and stress shared almost similar symptomatology, they are however different concepts. While stress is short-term and disappears once the situation becomes conducive, burnout is a long-term and complex phenomenon that gradually develops over an extended period of time (9, 65). Of note, untreated and unaddressed burnout may lead to personal chronic health consequences such as heart disease, stroke, obesity, or mental health consequences such as depressions, anxiety, substance and chemical use, and suicide (1, 66–71). Professionally, it may lead to reduced professional accomplishment and satisfaction (1, 66). Other closely related concepts (but with overlapping features) such as compassion fatigue, moral distress, and empathy fatigue captured different aspects of burnout (5). In general, this study provides background information for further research in this field, especially in surgical oncology.

### Artificial Intelligence as an Intervention to Mitigate Burnout in Oncology

With advancements in technology, the application of AI in medicine continues to grow significantly. In terms of mitigating burnout, AI is thought to be poised to offer a potential to mitigate burnout in oncology by enhancing the productivity and performance of the oncologists, reduce the workload and provide job satisfaction, and foster teamwork between the caregivers of patients with cancer (72).

There are arrays of studies that have been published regarding the potential of machine learning and AI for the prognostication of cancer which suggested that the performance and productivity of oncologists can be improved (72). For example, the prediction of recurrences and overall survival (73–75). Thus, AI technology such as deep learning is poised to enhance precision medicine (76–79) and improved clinical decisions (73, 74). With the improved clinical decisions, the oncologists may experience emotional satisfaction, reduced depersonalization, and increased professional efficacy. Hence, the it offers the potential to increase job satisfaction and reduce burnout of oncologists (80, 81).

In addition, the high workload has been reported as an important factor that contributes to occupational stress (82, 83). This has a negative effect on the quality of care offered to the patients. It has been reported that administrative tasks contribute to the workload of clinicians and significantly limit the time for direct clinical face time between the clinicians and patients (84). For example, it was found that physicians spend 49% of their work time on administrative tasks (desk work and EHRs) and 33% of their work time on direct clinicians-patient interaction (84). Thus, AI has been touted to significantly reduce this administrative burden (72).

Natural language processing, a branch of AI offers the potential for detailed and informative summarization of EHRs. It offers cognitive systems that can interpret, augment, and transform free text contained in the EHR and clinical notes in a format that can be represented for computation (85). This might be an instrument to reduce the workload and stress for the oncologists in terms of the onerous tasks of navigating and engagement with the EHR. With the aid of natural language processing, the structured data fields can be autopopulated from the notes of clinicians (72). Similarly, it can assist in the proper querying of relevant data of patients and transcribe past patient encounters. For example, it was reported that transcription (voice-to-text) can enhance work time savings of 17% for doctors (72).

Finally, the use of AI may aid through the optimized billing codes, and quality outcome reporting for hospital records and regulatory purposes (85). It also has the potential to integrate unstructured and structured data from different sources (72). This provides more cohesive, faster, and convenient access to information of patients across the multidisciplinary team in the oncological unit. This can greatly foster teamwork, easy collaboration, strong communication for shared decision making, and coordinated actions on the evaluation of the progress of the patients by the caregivers of patients with cancer (72). Therefore, the aforementioned benefits of AI intervention have the potential to further reduce workload and create a relaxed working environment for the oncologist and thus avoid stress. With these AI-based interventions, the oncologists may be well-positioned to perform with greater efficiency, engagement, and effectiveness (85).

## Conclusion

In conclusion, many oncological wards would have to deal with increasing admission of patients with cancer. This might be even more emphasized due to the recent outbreak of coronavirus pandemic. It is expected that work will become increasingly stressful and the possibility of burnout remains high. Therefore, healthcare management should recognize and seek to address this problem in oncology. Finding ways to ameliorate burnout is important for creating a conducive working environment for oncologists and to increase the well-being of oncologists and reduce medical errors. The interventions presented in this study will not achieve the required touted objectives without recognition of burnout as a problem by the healthcare institutions and professional bodies. This ensures that individual interventions are considered important, and consequently combinatorial strategies that include other interventions presented in this study offer the most viable hope to mitigating burnout among oncologists and positively affect the delivery of cancer care.

### Clinical Implications

The physical and emotional well-being of the clinical oncologists is important to enhance the quality of care, patient satisfaction, and overall success of the organizations where the oncologists work (5). The physical and mental distress resulting from burnout adversely affects clinical oncologists-patients relationships (51), poorer patient outcomes (40), increases inefficacy, errors, chronic health conditions, and decreased productivity of the clinical oncologists (5, 51). Consequently, their personal engagements outside work such as family life and lifestyles are usually affected (5, 13). Due to the increasing prevalence of burnout in oncology, some of the oncologists have tragically left their practice or retired early (after decades of specialized training) (86, 87) or even committed suicide in some cases (60). Thereby, an increasing shortage of oncologists is evident. Based on these implications, the oncologists need to recognize the stress and burnout symptoms, acquire mindfulness training, learn resilience and cognitive-behavioral psychotherapy skills, reiterate their professional objectives, and improve communications and interpersonal skills to ensure the quality of care offered to the patients. The burnout interventions listed in this study are targeted at effective wellness strategies for the clinical oncologists and to improve their professional environment.

### Strength and Limitations

Strengths of this study include: (a) a systematic scoping review of all the published studies that have examined burnout intervention in clinical oncology. It maps the current state of research in this subject area (oncology medical profession) and aids in future planning of research; (b) these interventions were broadly categorized into seven divisions, namely, individual, organizational, educational, team-based, art and entertainment, motivational, and disruptive technology; and (c) furthermore, the possibility of disruptive technology such as AI and its subfield, machine learning as a possible intervention for addressing burnout in a medical profession was examined. There are certain limitations relating to this scoping review. First, the quality assessment score of all the included studies was not significantly high. Additionally, there are a few concerns about some of the interventions mentioned in the articles included in this study. Most of these interventions were thought of as those that were targeted at either oncologists (individuals) or the organization. Rarely were the interventions aimed at targeting both the oncologists and the organizations (88). Considering interventions (how to address burnout) that target both individuals and organizations may be pertinent for an effective burnout reduction strategy. Of note, it is important to include more participants (oncologists) in the evaluation of the benefits of these interventions to evaluate their efficacy. Moreover, some of these interventions require significant evidence-based support and strategies needed for institutions to introduce them to their organizational structures. Thus, it is important to avoid generalizing the reviewed interventions. Also, some challenges must be addressed by policymakers, healthcare providers, industry, and patients before AI can safely be used in an oncological unit.

## Author Contributions

RA and AM: study concepts and study design. RA and OY: studies extraction. PH and AA: acquisition and quality control of included studies. RA, ME, AA, AM, and PH: data analysis and interpretation. RA, OY, AA, AM, and PH: manuscript preparation. AA, ME, and PH: manuscript review. AA, RA, and OY: manuscript editing. All authors approved the final manuscript for submission.

## Conflict of Interest

The authors declare that the research was conducted in the absence of any commercial or financial relationships that could be construed as a potential conflict of interest.

## Publisher's Note

All claims expressed in this article are solely those of the authors and do not necessarily represent those of their affiliated organizations, or those of the publisher, the editors and the reviewers. Any product that may be evaluated in this article, or claim that may be made by its manufacturer, is not guaranteed or endorsed by the publisher.
