# Supplementary material for: Mitigating Burnout in an Oncological Unit: A Scoping Review
Source: Front Public Health. 2021 Oct 1;9:677915. doi: 10.3389/fpubh.2021.677915 (PMC8517258; doi:10.3389/fpubh.2021.677915)
Supplement: Supplementary file 1 [file Table_1.docx]

Supplementary Table 1. Preliminary quality assessment of the included studies

| Studies | Design | Measurement | Intervention | Statistical analysis | Score (%) |
| --- | --- | --- | --- | --- | --- |
| Le Blanc et al., 2007 | 🗹 | 🗹 | 🗹 | ⌧ | 3 (75) |
|  |  |  |  |  |  |
| Italia et al., 2008 | 🗹 | 🗹 | 🗹 | ⌧ | 3 (75) |
|  |  |  |  |  |  |
| Bar-Sela et al., 2012 | 🗹 | 🗹 | 🗹 | ⌧ | 3 (75) |
|  |  |  |  |  |  |
| Moody et al., 2013 | 🗹 | 🗹 | 🗹 | ⌧ | 3 (75) |
|  |  |  |  |  |  |
| Mukherjee et al., 2014 | 🗹 | 🗹 | 🗹 | 🗹 | 4 (100) |
|  |  |  |  |  |  |
| Rasmussen et al., 2016 | 🗹 | 🗹 | 🗹 | 🗹 | 4 (100) |
|  |  |  |  |  |  |
| He et al., 2017 | 🗹 | 🗹 | 🗹 | 🗹 | 4 (100) |
|  |  |  |  |  |  |
| Kavalieratos et al., 2017 | 🗹 | ⌧ | 🗹 | ⌧ | 2 (50) |
|  |  |  |  |  |  |
| Vetter at al., 2018 | 🗹 | 🗹 | 🗹 | 🗹 | 4 (100) |
|  |  |  |  |  |  |
| Richardson et al., 2019 | 🗹 | 🗹 | 🗹 | 🗹 | 4 (100) |
|  |  |  |  |  |  |
| Kaimal et al., 2019 | 🗹 | 🗹 | 🗹 | ⌧ | 3 (75) |
|  |  |  |  |  |  |
| Weintraub et al., 2019 | 🗹 | 🗹 | 🗹 | ⌧ | 3 (75) |
|  |  |  |  |  |  |
| Royce et al., 2019 | 🗹 | ⌧ | 🗹 | 🗹 | 3 (75) |
|  |  |  |  |  |  |
| LeNoble et al., 2020 | 🗹 | ⌧ | 🗹 | ⌧ | 2 (50) |
|  |  |  |  |  |  |
| Turner et al., 2020 | 🗹 | 🗹 | 🗹 | ⌧ | 3 (75) |
|  |  |  |  |  |  |
| Abusanad et al., 2021 | 🗹 | 🗹 | 🗹 | ⌧ | 3 (75) |
|  |  |  |  |  |  |
| Mascaro et al., 2021 | 🗹 | 🗹 | 🗹 | 🗹 | 4 (100) |
